# Synthesis of a 1-Aryl-2,2-chlorosilyl(phospha)silene Coordinated by an *N*-Heterocyclic Carbene †

**DOI:** 10.3390/molecules21101309

**Published:** 2016-09-30

**Authors:** Andreas Wolfgang Kyri, Paresh Kumar Majhi, Takahiro Sasamori, Tomohiro Agou, Vitaly Nesterov, Jing-Dong Guo, Shigeru Nagase, Norihiro Tokitoh, Rainer Streubel

**Affiliations:** 1 Institute for Chemical Research, Kyoto University, Gokasho, Uji, 611-0011 Kyoto, Japan; kyri@uni-bonn.de (A.W.K.); pmajhi@boc.kuicr.kyoto-u.ac.jp (P.K.M.); tomohiro.agou.mountain@vc.ibaraki.ac.jp (T.A.); v.nesterov@tum.de (V.N.); guo_jd@boc.kuicr.kyoto-u.ac.jp (J.-D.G.); 2 Institut für Anorganische Chemie der Rheinischen Friedrich-Wilhelms-Universität Bonn, Gerhard-Domagk-Str. 1, 53121 Bonn, Germany; 3 Fukui Institute for Fundamental Chemistry, Kyoto University, 606-8103 Kyoto, Japan; nagase@ims.ac.jp

**Keywords:** main group elements, phosphasilene, *N*-heterocyclic carbene

## Abstract

Phosphasilenes, P=Si doubly bonded compounds, have received considerable attention due to their unique physical and chemical properties. We report on the synthesis and structure of a chlorophosphasilene coordinated by an *N*-heterocyclic carbene (NHC), which has the potential of functionalization at the Si–Cl moiety. Treatment of a silylphosphine, ArPH–SiCl_2_R_Si_ (Ar = bulky aryl group, R_Si_ = Si(SiMe_3_)_3_) with two equivalents of Im-Me_4_ (1,3,4,5-tetramethylimidazol-2-ylidene) afforded the corresponding NHC-coordinated phosphasilene, ArP=SiClR_Si_(Im-Me_4_) as a stable compound. Bonding properties of the P=Si bond coordinated to an NHC will be discussed on the basis of theoretical calculations.

## 1. Introduction

**Scheme 1:**
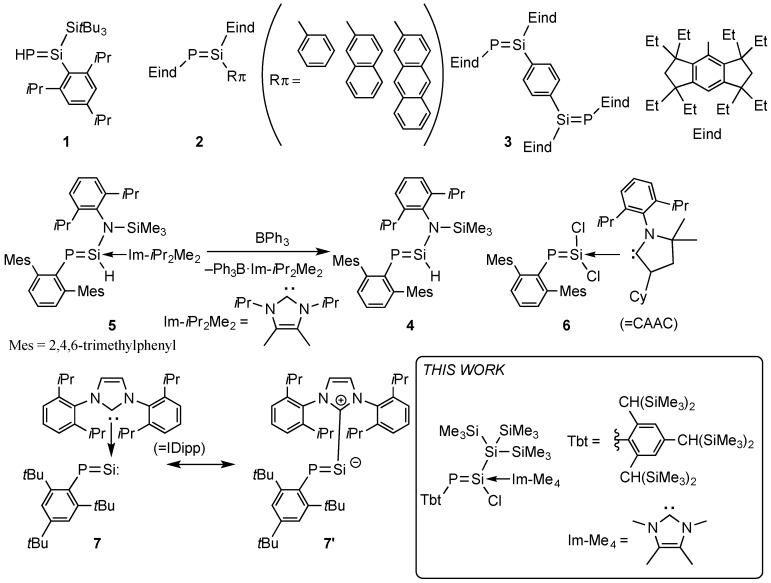
Examples of stable functionalized phosphasilenes and *N*-heterocyclic carbene (NHC)-coordinated phosphasilenes.

There has been much interest in the chemistry of multiple bond compounds between heavier main group elements due to their unique π-electron systems different from those of second row elements such as olefin, imine, and azo compounds. Several homonuclear multiple bond compounds between heavier main group elements have been synthesized as stable compounds by utilizing sterically demanding substituents attached to the reactive π-bond moiety [1]. Since the first isolation of a stable phosphasilene, a Si=P compound, reported by Bickelhaupt in 1984 [2], heteronuclear multiple bond compounds have also attracted many chemists because of their expected unique characters different from those of homonuclear systems [1,3,4]. Especially, phosphasilenes are of great interest because they can act as a strong π-accepting [5,6,7,8,9,10,11] ligands for transition metals. Up to now, several phosphasilenes have been synthesized as stable compounds including a “half-parent” phosphasilene, HP=Si(Tip)(Si*t*Bu_3_) (**1**) by Driess et al. [12]. Thus, phosphasilenes have generated much interest as heteronuclear π bond compounds between heavier group 14 and 15 elements in the expectation of their variety of electronic, optical, and magnetic properties when they are “functionalized” by further functional groups [13,14,15,16,17,18,19,20]. In this context, Tamao et al. have reported the synthesis of π-functionalized phosphasilenes with organic π-conjugated systems (**2**, **3**) and their Au complexes (Scheme 1) [21,22]. From these point of view, a proper phosphasilene synthon as a “building block” has been desired for synthetic studies of “functionalized” P=Si compounds. 

As a possible “building block” for a phosphasilene, some “functionalized” phosphasilenes have been reported. In general, such multiple bond compounds between heavier main group elements are highly reactive, accordingly they are difficult to isolate as stable compounds if they have a functional group instead of a sterically demanding group. In most cases, such functional phosphasilenes have been isolated as a donor–coordinated phosphasilene synthon bearing a strong donor such as an *N*-heterocyclic carbene (NHC) [23]. Functional phosphasilenes are interesting targets because, for example, the above-mentioned “half parent” phosphasilene **1** [5,6,7,8,9,10,11] has a PH moiety, which should be a nucleophilic building block of the P=Si moiety because of the lone pair of the P atom and its H^+^–P^−^ polarity. *H*,*N*-Functionalized phosphasilene **4** was isolated by Cui et al. [24], where the *H*,*N*-functionality afforded nucleophilicity at the P moiety. It should be noted that **4** was generated by the addition of BPh_3_ to the NHC-coordinated analogue **5**. Thus, it can be concluded that an NHC-coordinated phosphasilene should be an appropriate precursor under mild conditions. In addition, Roesky et al. reported cyclic(amino)(alkyl)carbene (CAAC)-coordinated 2,2-dichlorophosphasilene **6** [25], which was described as a carbene-dichlorosilylene stabilized phosphinidene. Moreover, it would be expected to behave as an electrophilic phosphasilene building block synthon because of the Si–Cl moiety, though it would be difficult to remove the CAAC moiety because of its high σ-donating ability [26]. One of the conceivable promising “building blocks” for a phosphasilene should be a phosphasila-vinylidene analogue, RP=Si: which would exhibit an amphiphilic, nucleophilic and electrophilic, character because of its P=Si: moiety. Recently, Filippou et al. reported the synthesis of the NHC-coordinated phosphasila-vinylidene derivative **7** [27], which was synthesized by the reaction of IDipp-SiCl_2_ (IDipp = 1,3-bis(2,6-diisopropylphenyl)-imidazol-2-ylidene) [28] with Mes*P(SiMe_3_)Li (Mes* = 2,4,6-tri-*t*-butylphenyl) [29] via 1,2-elimination of Me_3_SiCl [30,31,32]. Because of the strong σ-coordination of IDipp, **7** exhibits not a silavinylidene character but the corresponding silyl anion character **7′** which has an isoelectronic structure to a diphosphene. Based on these considerations, we have designed, and report hereafter, a silyl-chloro substituted phosphasilene representing a phosphasilenylidene synthon because of its potential α-silylchloride elimination.

## 2. Results and Discussions

**Scheme 2:**
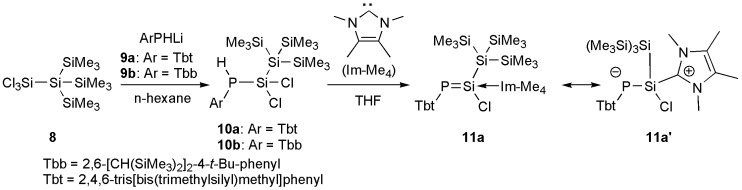
Synthesis of NHC-coordinated phosphasilene **11a**.

Treatment of silyl-substituted trichlorosilane **8**, (Me_3_Si)_3_Si–SiCl_3_ [33], with ArPHLi (**9**, Ar = Tbt or Tbb, Tbt = 2,4,6-tris[bis(trimethylsilyl)methyl]phenyl, Tbb = 2,6-bis[bis(trimethylsilyl)methyl]-4-*t*-butylphenyl) [34] afforded the corresponding dichloro(phosphino)silanes **10a**,**b** (Scheme 2). The reaction of **10a** with MN(SiMe_3_)_2_ (M = Li, Na, K) as base that was expected to generate the corresponding phosphasilene ArP=Si(Cl)[Si(SiMe_3_)_3_], resulted in no observable reaction. In contrast, treatment of **10a** with *n*- or *t*-butyllithium gave a complicated and unidentifiable product mixture. When **10a** was treated with Im-Me_4_ (1,3,4,5-tetramethylimidazol-2-ylidene) [35], the NHC-coordinated 1-aryl-2,2-chlorosilylphosphasilene **11a** was obtained as a stable orange-red compound. The ^31^P-NMR spectra (C_6_D_6_) of **11a** showed singlet signals in the upper-field region (δ_P_ = −117), indicating a considerable contribution of phosphanide character represented by the canonical structure **11a**′. 

**Figure 1:**
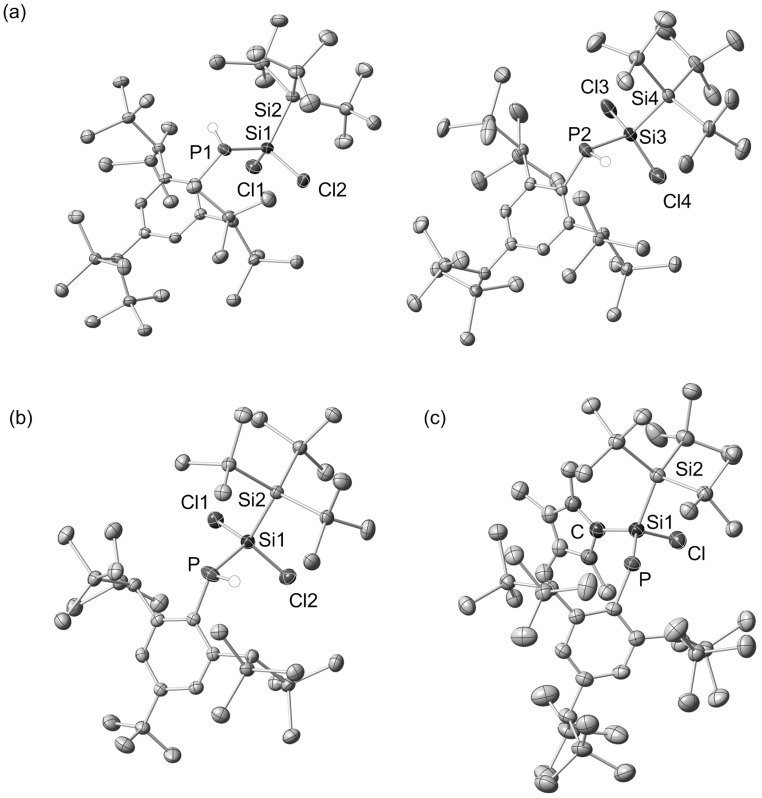
Molecular structures of (**a**) **10a** (two independent molecules in the unit cell); (**b**) **10b**; and (**c**) **11a** with atomic displacement parameters set at 50% probability. All hydrogen atoms other than those at the P atoms are omitted for clarity and only selected atoms are labeled. Selected bond lengths (Å) (a) **10a**: P1–Si1, 2.2463(8); Si1–Si2, 2.3318(8); Si1–Cl1, 2.0636(8); Si1–Cl2, 2.0853(8) (molecule A); P2–Si3, 2.2465(8); Si3–Si4, 2.3370(8); Si3–Cl3, 2.0764(9); Si3–Cl4, 2.0699(9) (molecule B). (b) **10b**: P–Si1, 2.2281(8); Si1–Si2, 2.3203(7); Si1–Cl1, 2.0691(7); Si1–Cl2, 2.0672(7). (c) **11a**: Si1–P, 2.1319(11); Si1–Cl, 2.1907(11); Si1–Si2, 2.3707(11); Si1–C, 1.1949(3). Numbers in parenthesis correspond to the standard deviation of the values.

The structural parameters in the crystalline states of dichloro(phosphino)silanes **10a**,**b** and NHC-coordinated phosphasilene **11a** were determined by the X-ray crystallographic analysis (Figure 1) [36]. The Si–P bond length in **11a** [2.1319(11) Å] is apparently shorter than those in the precursors **10a**,**b** [2.2463(8), 2.2465(8) for **10a**, 2.2281(8) for **10b**] and typical Si–P single bonds. This structural feature suggested some degree of P–Si multiple bond character in **10a** despite its Si atom is already saturated due to the Si–P, Si–Si, Si–Cl, and Si–C single bonds. The detailed bond character of P–Si bond in **11a** has been revealed on the basis of theoretical calculations.

**Scheme 3:**
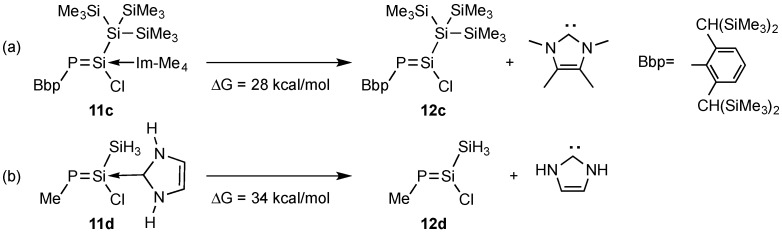
Theoretical calculations of NHC-coordinated and non-coordinated phosphasilenes. (**a**) B3PW91-D3BJ/6-31+G(d,p)//B3PW91/6-31+G(d,p); (**b**) MP2/6-311G(2d).

**Figure 2:**
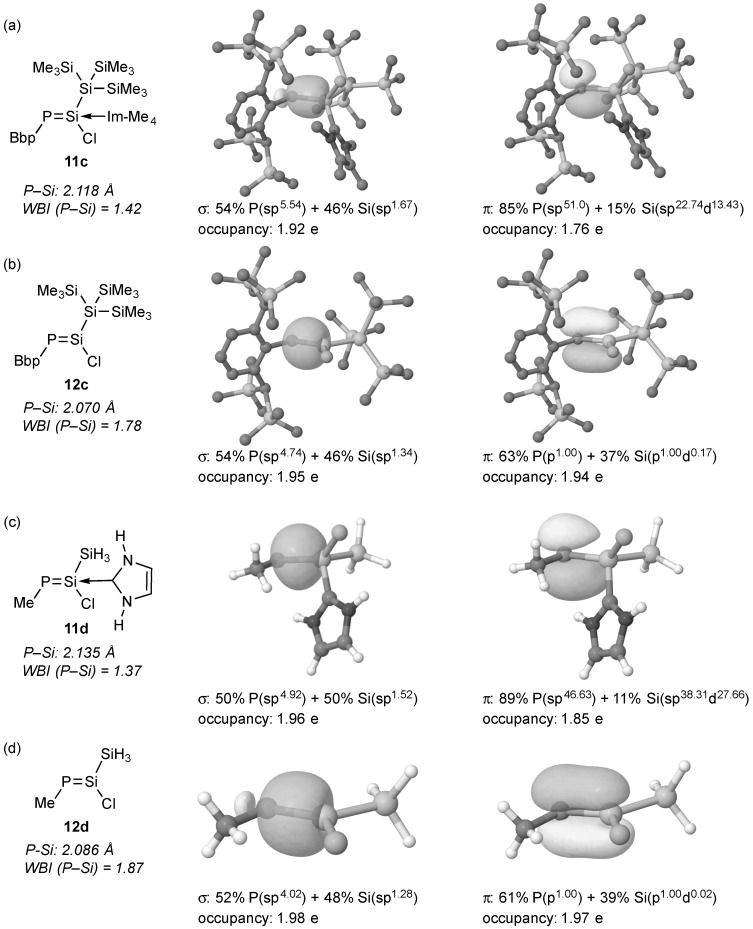
NBO (Natural Bond Orbital) calculations for NHC-coordinated and unligated phosphasilenes. (**a**,**b**) B3PW91/6-31+G(d,p)//B3PW91/3-21G(d), (**c**,**d**) MP2/6-311G(2d). WBI, Wiberg bond index.

Optimized structural parameters of **11c** at B3PW91/6-31+G(d,p) level, which has a Bbp group (2,6-[CH(SiMe_3_)_2_]_2_-C_6_H_3_) instead of the Tbt group of **11a** were found to be in good agreement with those experimentally observed by X-ray crystallographic analysis. NBO (Natural Bond Orbital) calculations [37] of **11c** showed two chemical bonds between the P and Si atoms, indicating the existence of a P=Si π bond. The first P–Si bond is σ-type bonding, 54% P (sp^5.54^) + 46% S (sp^1.67^), suggesting its typical Si–P σ-bond character. The second one is π-type bonding, 85% P (sp^51.0^) + 15% Si (sp^22.74^d^13.43^). The predominant contribution to this chemical bond seems to be caused by the coordination of the lone pair of P (3p orbital) to the vacant 4d orbital of the Si atom. As it can be deduced from the NBO values (Figure 2), this π-bond is rather weak, i.e., it is mostly localized on the P atom. The latter also reveals the shielding of the nucleus and, hence, provides some explanation for the highfield shifted ^31^P resonance relative to **11a**; this also lends support to the predominant contribution of the canonical structure of **11a′**. However, the Wiberg bond index (WBI) of the P–Si bond was computed as 1.42, showing its multiple bond character to some extent. This bonding character should not arise from the steric hinderance exerted by the bulky aryl group and silyl group, because the less hindered model compound **11d** was found to exhibit bonding characters similar to those of **11c**. That is, the P–Si bond in **11d** was composed of σ- and π-type NBOs, 50% P (sp^4.92^) + 50% Si (sp^1.52^) and 89% P (sp^46.43^) + 11% Si (sp^38.31^d^27.66^), where the WBI of Si–P bond is 1.37. It can be expected that such π-bonding character between the Si and P atoms would promote the elimination of the Im-Me_4_ moiety from **11a** to give the corresponding donor free phosphasilene with the Si=P double bond. Indeed, the dissociation of the Si–Im-Me_4_ coordination in **11c** to give the corresponding phosphasilene **12c** and Im-Me_4_ were computed as ΔG = +28 kcal/mol [38,39] while the Si–NHC dissociation of the less hindered model **11d** to give **12d** was highly endothermic (ΔG = +34 kcal/mol), suggesting that the steric demand would reduce the dissociation energy of the Si–NHC coordination (Scheme 3) [40]. Regarding the ideally generated phosphasilene **12c**, it was confirmed that it exhibits considerable π-bond character of the P=Si bond on the basis of NBO calculations. That is, the P=Si bond in **12c** was composed of σ- and π-bonds, 54% P (sp^4.74^) + 46% Si (sp^1.34^) (σ-bond) and 63% P (p^1.00^) + 37% Si (p^1.00^d^0.17^) (π-bond), with WBI = 1.78. At the same time **12d**, the less hindered model, exhibits similar P=Si bonding properties with WBI = 1.87, 52% P (sp^4.02^) + 48% Si (sp^1.28^) (σ-bond) and 61% P (p^1.00^) + 39% Si (p^1.00^d^0.02^) (π-bond), indicating that the sterically demanding substituents would not perturb the P=Si bonding characters in the phosphasilenes.

## 3. Materials and Methods

### 3.1. General Information

All manipulations were carried out under an argon atmosphere using either Schlenk line techniques or glove boxes. Solvents were purified by the Ultimate Solvent System, Glass Contour Company (Nikko Hansen & Co., Osaka, Japan) [41]. ^1^H-, ^13^C-, ^28^Si-, and ^31^P-NMR spectra were measured on a JEOL AL-300 spectrometer (JEOL Ltd., Tokyo, Japan) or a Bruker Avance-600 spectrometer (Bruker, Billerica, MA, USA). High-resolution mass spectra (HRMS) were measured on a Bruker micrOTOF focus-Kci mass spectrometer (on ESI-positive mode) (Bruker). Elemental analysis was carried out by using Micro Corder JM10 (J-Science Lab Co., Ltd., Kyoto, Japan) at the Microanalytical Laboratory, Institute for Chemical Research, Kyoto University. TMS_3_SiSiCl_3_ was prepared according to literature procedure [33].

### 3.2. Synthesis

#### 3.2.1. General Procedure for the Synthesis of Phosphinodichlorosilanes **10a** and **10b**

To a solution of TbtPH_2_ or TbbPH_2_ (0.6 mmol each) in 5 mL diethyl ether was added 0.412 mL of *n*-BuLi (0.66 mmol, 1.6 M in *n*-hexane) at ambient temperature. The reaction mixture was stirred for 1 h followed by the addition of 229.2 mg (0.6 mmol) of (Me_3_Si)_3_SiSiCl_3_ in 5 mL of *n*-hexane. The solution was further stirred for 6 h. After the reaction was completed (^31^P-NMR check), all volatiles were removed in vacuo (7 × 10^−3^ mbar). The crude mixture was dissolved in *n*-hexane and filtered over celite to remove the inorganic salt. The crude product was recrystallized (in the case of **10a**) by slow evaporation of an *n*-hexane solution at ambient temperature.

Data for **10a**: Yield = 78.8 mg (0.083 mmol, 14%), white solid. ^1^H-NMR (600 MHz, C_6_D_6_, 28 °C): δ = 0.18 (s, 18H, SiMe_3_), 0.31 (s, 18H, SiMe_3_), 0.34 (s, 18H, SiMe_3_), 0.42 (s, 27H, Si(SiMe_3_)_3_), 1.48 (s, 1H, *para*-C*H*(SiMe_3_)_2_), 2.69 (d, 1H, ^4^*J*_P,H_ = 2.4 Hz, C*H*(SiMe_3_)_2_), 2.79 (d, 1H, ^4^*J*_P,H_ = 5.4 Hz, C*H*(SiMe_3_)_2_), 4.46 (d, 1H, ^1^*J*_P,H_ = 224.3 Hz, P-*H*), 6.66 (s, 1H, C_6_H_5_-*H*), 6.66 (s, 1H, C_6_H_5_-*H*). ^13^C{^1^H} NMR (151 MHz, C_6_D_6_, 28 °C): δ = 1.0 (s, SiMe_3_), 1.7 (s, SiMe_3_), 1.8 (s, SiMe_3_), 2.6 (s, Si(SiMe_3_)_3_), 29.8 (d, ^3^*J*_P,C_ = 6.1 Hz, *C*H(SiMe_3_)_2_), 30.1 (d, ^3^*J*_P,C_ = 11.3 Hz, *C*H(SiMe_3_)_2_), 30.9 (s, *para*-*C*H(SiMe_3_)_2_), 121.3 (d, ^1^*J*_P,C_ = 17.7 Hz, P-*C*), 122.9 (s, Ar), 128.3 (s, Ar), 143.8 (s, Ar), 150.1 (d, ^2^*J*_P,C_ = 12.1 Hz, Ar), 150.7 (d, ^2^*J*_P,C_ = 17.3 Hz, Ar). ^29^Si{^1^H} NMR (119.2 MHz, C_6_D_6_, 28 °C): δ = −8.9 (s, Si(*Si*Me_3_)_2_), 2.2 (s, CH(*Si*Me_3_)_2_), 3.2 (s, CH(*Si*Me_3_)_2_), 39.5 (d, ^1^*J*_P,Si_ = 110.7 Hz, P-*Si*Cl_2_); central Si-atom of *Si*(SiMe_3_)_3_ was not observed. ^31^P{^1^H} NMR (253 MHz, C_6_D_6_, 28 °C): δ = −113.5 (s_sat_, ^1^*J*_P,Si_ = 110.7 Hz). HRMS: *m*/*z* calcd. for C_36_H_87_Cl_2_PSi_11_+H^+^, 931.3444; found, 931.3488. Elemental analysis: calcd. for C_36_H_87_ Cl_2_PSi_11_: C 46.45, H 9.42 found: C 45.62, H 9.29. 

Data for **10b**: The ^31^P-NMR of the reaction mixture revealed the formation of two products [δ = –115.9 (^1^*J*_P,H_ = 223.4 Hz) (**10b**) and -147.8 (^1^*J*_P,H_ = 210.4 Hz)] in 85:15 ratio. The latter signal was identified to be TbbP(H)SiMe_3_. Compound **10b** precipitated in small amounts only after keeping the crude mixture in *n*-hexane solution over three weeks at −20 °C; the crystals thus obtained were used for the X-ray diffraction study. 

#### 3.2.2. Synthesis of NHC-coordinated Phosphasilene **11a**

A quantity of 93 mg (0.1 mmol) of **10a** was dissolved in 3 mL of THF and a solution of 25 mg (0.2 mmol) of 1,3,4,5-tetramethyl-imidazol-2-ylidene in 3 mL of THF was added while stirring at ambient temperature. During the addition a solid precipitate was observed and the color turned to orange-red. The solution was stirred for additional 15 min before all volatiles were removed in vacuo (7 × 10^−3^ mbar). The crude mixture was dissolved in *n*-hexane and filtered over celite to remove imidazolium salt. After keeping the *n*-hexane solution at r.t. for one day of the crude mixture some crystals precipitated of which X-ray diffraction and NMR analysis was performed.

Data for **11a**: Orange-red solid. ^1^H-NMR (600 MHz, toluene-*d*_8_, 28 °C): δ = 0.14 (s, 18H, SiMe_3_), 0.21 (d, 18H, ^6^*J*_P,H_ = 1.7 Hz, SiMe_3_), 0.40 (s, 18H, SiMe_3_), 0.47 (s, 27H, Si(SiMe_3_)_3_), 1.35 (s, 6H, N-Me), 1.43 (s, 1H, *para*-C*H*(SiMe_3_)_2_), 3.64 (s, 6H, C-Me), 4.09 (s, 1H, C*H*(SiMe_3_)_2_), 4.16 (s, 1H, C*H*(SiMe_3_)_2_), 6.58 (s, 1H, C_6_H_5_-*H*), 6.70 (s, 1H, C_6_H_5_-*H*). ^13^C{^1^H} NMR (151 MHz, toluene-*d*_8_, 28 °C): δ = 1.2 (d, ^5^*J*_P,C_ = 3.0 Hz, SiMe_3_), 1.5 (s, SiMe_3_), 1.7 (s, SiMe_3_), 2.0 (s, SiMe_3_), 2.2 (s, SiMe_3_), 3.8 (d, ^4^*J*_P,C_ = 3.5 Hz, Si(SiMe_3_)_3_), 8.1 (s, C-Me), 29.6 (s, *C*H(SiMe_3_)_2_), 29.9 (s, *C*H(SiMe_3_)_2_), 30.1 (s, *para*-*C*H(SiMe_3_)_2_), 36.0 (s, N-Me), 121.8 (s, C=C), 126.4 (s, Ar), 137.7 (d, ^1^*J*_P,C_ = 69.3 Hz, P-*C*), 137.9 (d, *J*_P,C_ = 1.1 Hz, Ar),150.7 (s, Ar), 153.9 (s, C^2^). ^29^Si{^1^H} NMR (119.2 MHz, toluene-*d*_8_, 28 °C): δ = −111.9 (d, ^2^*J*_P,Si_ = 47.6 Hz, *Si*(SiMe_3_)_2_), −22.4 (d, ^1^*J*_P,Si_ = 225.5 Hz, P-*Si*Cl(Im-Me_4_)), −9.1 (d, ^3^*J*_P,Si_ = 8.4 Hz, Si(*Si*Me_3_)_3_), 1.5 (s, CH(*Si*Me_3_)_2_), 1.8 (s, CH(*Si*Me_3_)_2_), 2.1 (s, CH(*Si*Me_3_)_2_). ^31^P{^1^H} NMR (253 MHz, toluene-*d*_8_, 28 °C): δ = −117.1 (s_sat_, ^1^*J*_P,Si_ = 225.5 Hz). HRMS: *m*/*z* calcd. for C_43_H_98_ClN_2_PSi_11_ + H^+^, 1019.4685; found, 1019.4689.

### 3.3. Computational Methods

The level of theory and the basis sets used for the structural optimization are contained within the main text. Frequency calculations confirmed minimum energies for all optimized structures. All calculations were carried out using the *Gaussian 09* Rev. D.01 (Gaussian, Inc., Wallingford, CT, USA) program package. Computational time was generously provided by the Supercomputer Laboratory in the Institute for Chemical Research of Kyoto University.

### 3.4. X-ray Crystallographic Analysis

Single crystals of **10a**, [**10b**·C_6_H_14_], and **11a** were obtained from recrystallization from *n*-hexane. Intensity data were collected on a RIGAKU Saturn70 CCD system with VariMax Mo Optics (Rigaku, Tokyo, Japan) using Mo Kα radiation (λ = 0.71075 Å). Crystal data are shown in the references. The structures were solved by a direct method (SIR2004 [42]) and refined by a full-matrix least square method on *F*^2^ for all reflections (SHELXL-97 [43]). All hydrogen atoms were placed using AFIX instructions, while all other atoms were refined anisotropically. Supplementary crystallographic data were deposited at the Cambridge Crystallographic Data Centre (CCDC; under reference numbers: CCDC–1501067, 1501065, and 1501066 for **10a**, [**10b**·C_6_H_14_], and **11a**, respectively). Copies of the data can be obtained, free of charge, via www.ccdc.cam.ac.uk/data_request.cif (accessed on 26 August 2016).

## 4. Conclusions

The NHC-adduct of chlorosilylphosphasilene has been synthesized and structurally characterized. It is evidenced that even the NHC-adduct **11a** exhibited P=Si multiple bond character in which, on the basis of theoretical calculations, a d-orbital of the Si atom is involved. Furthermore, the NHC-adduct **11a** should be a possible precursor for the corresponding “functionalized” phosphasilene **12a** because the dissociation of NHC is computed to be exothermic. Further investigations on the generation of the phosphasilenes **12a** and application of **10a** and **10b** towards utilization as a building block for P=Si moiety are currently in progress.
